# Aversive Learning in the Praying Mantis (*Tenodera aridifolia*), a Sit and Wait Predator

**DOI:** 10.1007/s10905-018-9665-1

**Published:** 2018-02-22

**Authors:** Thomas Carle, Rio Horiwaki, Anya Hurlbert, Yoshifumi Yamawaki

**Affiliations:** 1 Present Address: Department of Biology, Faculty of Science, Kyushu University, Fukuoka, 819-0395 Japan; 2 Present Address: Institute of Neuroscience, Newcastle University, Newcastle upon Tyne, NE2 4HH UK

**Keywords:** Avoidance behavior, bitter taste, learning, praying mantis, predator, prey

## Abstract

Animals learn to associate sensory cues with the palatability of food in order to avoid bitterness in food (a common sign of toxicity). Associations are important for active foraging predators to avoid unpalatable prey and to invest energy in searching for palatable prey only. However, it has been suggested that sit-and-wait predators might rely on the opportunity that palatable prey approach them by chance: the most efficient strategy could be to catch every available prey and then decide whether to ingest them or not. In the present study, we investigated avoidance learning in a sit-and-wait predator, the praying mantis (*Tenodera aridifolia*). To examine the effects of conspicuousness and novelty of prey on avoidance learning, we used three different prey species: mealworms (novel prey), honeybees (novel prey with conspicuous signals) and crickets (familiar prey). We sequentially presented the prey species in pairs and made one of them artificially bitter. In the absence of bitterness, the mantises consumed bees and crickets more frequently than mealworms. When the prey were made bitter, the mantises still continued to attack bitter crickets as expected. However, they reduced their attacks on bitter mealworms more than on bitter bees. This contrasts with the fact that conspicuous signals (e.g. coloration in bees) facilitate avoidance learning in active foraging predators. Surprisingly, we found that the bitter bees were totally rejected after an attack whereas bitter mealworms were partially eaten (~35%). Our results highlight the fact that the mantises might maintain a selection pressure on bees, and perhaps on aposematic species in general.

**Electronic supplementary material:**

The online version of this article (10.1007/s10905-018-9665-1) contains supplementary material, which is available to authorized users.

One of the key factors driving evolution is the relationship between prey and predators. On one hand, prey need to defend themselves, and different strategies have emerged such as camouflage, the use of toxins or other noxious compounds associated with warning signals (aposematism) or mimicry (for review, Ruxton et al. 2004). On the other hand, although predators have to ingest sufficient nutrients to fulfill their energy requirements, they have to avoid harmful prey and the ingestion of toxins that induce malaise and vomiting, and may lead to death. To this end, animals discriminate between different tastes (for reviews Rogers and Newland 2003; Yarmolinsky et al. 2009), and innately reject and avoid bitter tasting foods (Glendinning 1994).

In addition to gustatory cues, animals associate cues at a distance (e.g. olfactory, auditory and visual cues) with the palatability of food in order to make decisions about initiating predatory behaviour. In these associative processes, conspicuousness and novelty are known to facilitate avoidance learning (e.g. Shettleworth 1972; Gittleman and Harvey 1980; Gamberale-Stille 2001), which may explain the survival of aposematic species and their evolution (Wallace 1879; Cott 1940). Other species also “partially” benefit from this protection by mimicking these conspicuous patterns without paying the cost of secreting or stocking toxins (Cohen 1985; Rowell-Rahier and Pasteels 1986): Batesian mimicry (Bates 1861; Poulton 1890).

Although birds, because of their cognitive capabilities, have been intensively used until now to investigate such evolutionary questions, it is likely that avoidance-learning processes depend on predatory strategy: e.g. active foragers vs sit-and-wait foragers (see Huey and Pianka 1981; Pyke 1984; Beachly et al. 1995). Active foragers spend time and energy searching for prey and quickly decide to leave sites where the probability of finding food is low (Krebs et al. 1974; Brown 1988) to switch to sites where food is more abundant (see Pyke 1984). In this kind of strategy, efficiency in searching for prey is important for saving energy and facilitated by using distant cues.

In contrast, sit-and-wait predators do not invest much time and energy in searching for prey (e.g. Anderson and Karasov 1981), although they also switch to sites where prey are abundant (Morse 1986). In this case, the probability of finding and catching a prey mostly depends on the movement of the prey and not on that of the predator. For sit-and-wait predators that do not capture prey in a web, because of the uncertainty of encountering palatable prey, the most efficient strategy might be to catch every available prey and then decide whether to ingest or not (Malcolm 1986; Toft and Wise 1999). An example of this strategy is seen with wolf spiders (*Schizocosa* sp.) which catch both palatable (fruitflies) and unpalatable (fungus gnats) prey when they are offered alternately, and release unpalatable prey (Toft and Wise 1999). Therefore, sit-and-wait predators seem to exhibit distinct behaviours and cognitive processes compared to active foragers, yet relatively little is known about these processes (but see Prudic et al. 2007).

Using praying mantises (*Tenodera aridifolia*) as an example of a sit-and-wait predator, in the present study we tested their avoidance learning for natural conspicuous and for novel prey. By selecting natural prey, we used the following three species: crickets as familiar prey; mealworms as novel prey; and bees as conspicuously novel prey. Indeed, our choice is based on the fact that bees are referenced as an aposematic species in contrast to mealworms (Cott 1940). Therefore, it is likely that bees are visually more detectable and stimulating for mantises. The praying mantis visually detects prey and captures it with its forelegs (Roeder 1960; Yamawaki 2017) and their rate of attack is a good indicator of their feeding decisions. Therefore, we presented the different prey types in front of mantises by mechanically moving them at a constant speed to elicit mantis strikes. We tested the avoidance learning for each type of natural prey by also making each artificially unpalatable by injecting it and coating it with a bitter substance. We expected that, similarly to active foragers, the mantises would learn to avoid bitter prey that are novel and/or conspicuous faster than ones that were familiar. However, our results did not support this expectation: mealworms (novel) were avoided faster than bees (novel and conspicuous). Instead our results showed that visual conspicuousness did not improve avoidance learning in the mantis when the prey were made bitter, and that visual conspicuousness facilitates attacks in praying mantis.

## Methods

### Subjects and Housing

In total, 44 adult female praying mantises (*Tenodera aridifolia*) were used as predators. Males were excluded because of their variable foraging behavior and overall lower level of prey ingestion (e.g. Carle et al. 2015), compared to females. Oothecae were collected in a suburb of Fukuoka (Japan) on grassland near Tachibana mountain (+33° 40′ 46.7′′, +130° 28′ 6.20′′). The nymphs obtained were bred to adulthood using methods previously described (e.g. Sato and Yamawaki 2014; Carle et al. 2015). The mantises were kept at 25 ± 3 °C and in a 12 h:12 h L:D photoperiod (light phase, 9:00–21:00) during breeding. They were kept together in a plastic box (40 × 23 × 25 cm) provided with mesh walls inside for moulting and with aerations on the top. During breeding, they were fed with fruit flies (*Drosophila melanogaster*) three times per week, in addition to water ad libitum until the 3rd instar. Then, we provided nymphs of crickets (*Acheta domesticus,* ca. 5–20 mm lengths) at the same frequency. At this moment, individuals were isolated and placed in similar plastic boxes that were partitioned into nine compartments (13 × 7 × 25 cm), and water was sprayed at the top of cages after food was provided. At adulthood, each individual was placed in an individual box (15 × 10 × 20 cm) and received the same diet as previously. Prior to experiments, the mantises were food deprived for 3 days.

### Prey

Three different prey species were used: crickets (*Acheta domesticus,* ca. 10 mm in length) as familiar prey to the mantis, mealworms (*Tenebrio molitor* larvae, ca. 20 mm in length) as novel and relatively cryptic prey, and honeybee workers (*Apis mellifera*, 10 mm in length: their sting and associated venom gland were removed to avoid the natural toxins of bees) as conspicuously novel prey. The mealworms were chosen based on our previous work showing that the mantises stop eating mealworms injected with 500 mM of DB (Carle et al. 2015), and because they are less conspicuous than bees, to the mantis visual system (see below). All of these prey species were obtained from commercial suppliers. Each mantis only received two of these prey species as follow: cricket/worm, bee/worm or cricket/bee.

### Contrast of Prey

**Fig. 1:**
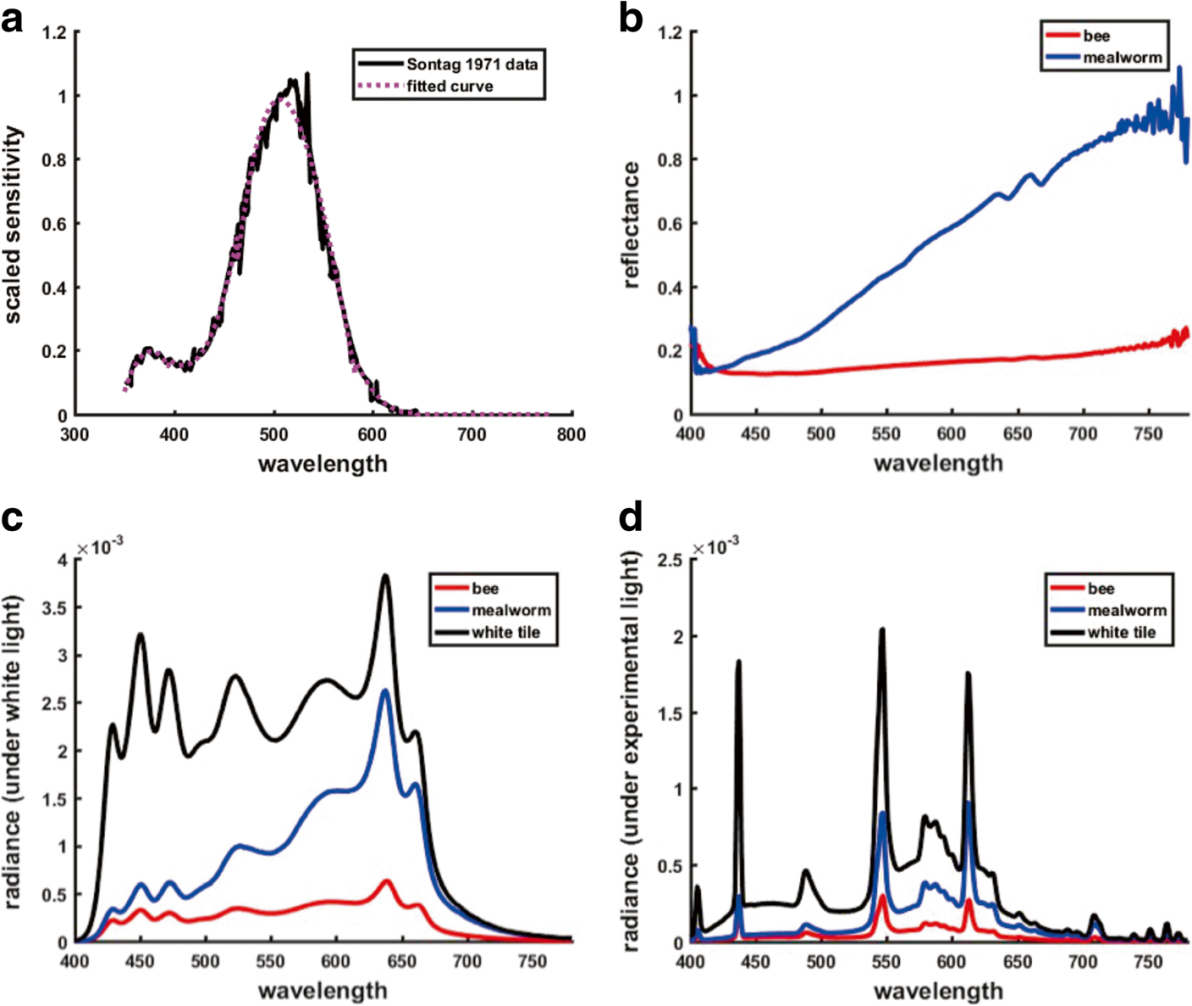
**Contrast of prey. a:** Spectral sensitivity of dark-adapted compound eye in mantis from Sontag (1971). **b:** Surface spectral reflectance of the darkest portion of bee (red) and mealworm (blue). **c, d:** Surface radiance of bee (red), mealworm (blue) and from the experimental whiteboard (black) under the experimental illumination **(c)** and under a broad-band white light **(d)**

The visual (Michelson) contrast of the prey, as seen through the mantis’s compound eye, was calculated as:

$$ contrast=\left({\mathrm{q}}_{prey}-{\mathrm{q}}_{board}\right)/\left({\mathrm{q}}_{prey}+{\mathrm{q}}_{board}\right) $$

where q_*prey*_ is the response of the photoreceptor in the mantis compound eye to the light reflected from the prey, under the experimental conditions, and q_*board*_ is the response to the whiteboard against which the prey is displayed. These responses are calculated using standard models for surface-light interactions and receptor activations (e.g. Wandell 1995; Hurlbert 1998; Kinoshita and Arikawa 2000; Fabricant and Herberstein 2014):

$$ {\displaystyle \begin{array}{l}{\mathrm{q}}_{prey}={\int}_{\lambda}\mathrm{s}\left(\uplambda \right){I}_{prey}\left(\uplambda \right)d\uplambda \\ {}{\mathrm{q}}_{board}={\int}_{\lambda}\mathrm{s}\left(\uplambda \right){I}_{board}\left(\uplambda \right)d\uplambda \end{array}} $$

where

$$ {I}_{prey}\left(\uplambda \right)={\mathrm{r}}_{prey}\left(\uplambda \right)E\left(\uplambda \right) $$

$$ {I}_{board}\left(\uplambda \right)={\mathrm{r}}_{board}\left(\uplambda \right)E\left(\uplambda \right) $$

and r_*prey*_(λ) corresponds to the surface spectral reflectance of the prey’s body; r_*board*_(λ) the surface spectral reflectance of the whiteboard; *E*(λ) the spectral power distribution of the illumination; *I*_*prey*_(λ) the surface spectral radiance reflected from the prey’s body (and similarly for the whiteboard). For the spectral sensitivity of the mantis visual receptors, s(λ), we used the sensitivity of the dark-adapted compound eye in mantis *Tenodera sinensis*, as provided in Sontag (1971) (Fig. 1).

Surface radiance measurements were made from a 0.2 degree spot centred on the prey’s body (for the bee, the spot was located on the darkest portion of the bee’s body), from a distance of approximately 50 cm (i.e. a spot less than 2 mm in diameter on the prey’s body), using a Konica Minolta CS-2000 spectroradiometer, under (1) the experimental illumination (positioned as in the experiment, at a distance of 50 cm above the board) and (2) a broad-band white light. Surface radiance measurements were made from the experimental whiteboard directly adjacent to the prey, under the same illumination conditions. Analogous measurements and definitions apply for both mealworm and bee.

The computed visual (Michelson) contrasts are −0.81 for the bee and −0.50 for the mealworm. Both contrasts are negative, i.e. the prey are darker than the background, and fall in the tracking and striking range (Prete et al. 2011), but the visual contrast of the bee is 1.63 times greater than that of the mealworm.

### Experimental Set-up

**Fig. 2:**
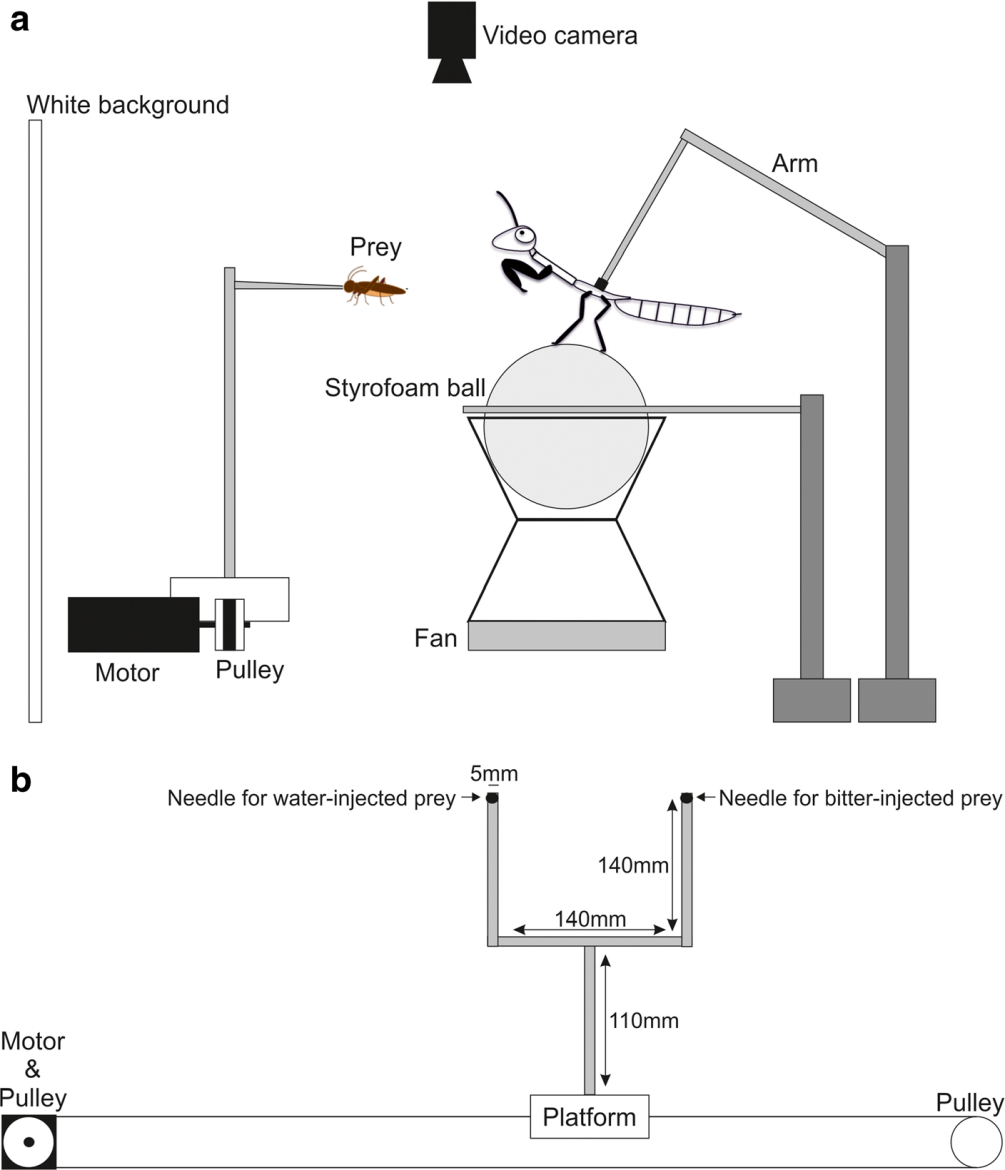
**Experimental set-up viewed from the side (a) and motorized system viewed from behind (b).** During experiments, the mantises were placed on a styrofoam ball and fixed to an arm. The prey was impaled with a needle on a platform. The platform held two needles, one for normal prey and the other for bitter prey, in order to avoid any taste contaminations. The platform was horizontally moved at a constant speed (205 mm/s) along rails by an electronic motor and pulleys

During the experiments, the mantises were tethered to an apparatus (Fig. 2a). A piece of pin header (2131D2*40GSE, Linkman, Japan) was stuck on the dorsal pterothorax of the mantises with beeswax, and a piece of pin socket (21602x40GSE, Linkman, Japan) was fixed on the terminal tip of a flexible arm. During the experiments, the mantises were tethered to the arm by inserting the pin header into the socket, and were positioned on a Styrofoam ball (12 cm in diameter) that was kept airborne by a small fan, allowing them leg movements. They were then given one hour to acclimate to the apparatus before starting the protocols.

During the protocols, because it has been shown that speed may affect mantises’ decision-making for attacking (e.g. Prete et al. 1993), the prey were mechanically moved leftward or rightward at a constant speed of 205 mm/s, by a custom-made apparatus modified from Yamawaki (2011), as this speed successfully elicit strikes in mantises (preliminary experiments). The prey was impaled with a needle onto a platform. After being impaled, we did not observe movements of the prey as we smashed the content of their abdomen and thorax with a needle before injecting them (see below). The platform held two needles, the one for normal prey and the other for bitter prey (Fig. 2b), in order to avoid any taste contaminations. Only one of these needles was used in a given presentation, and these needles were separated from each other (140 mm) to avoid the disturbance by the unused needle. The platform was horizontally moved along rails by an electronic motor (US206–401, Oriental Motor) and pulleys. The distance to prey was kept at 2 cm when the prey was located in front of the mantis, which was the optimal distance for the tethered mantises to capture the prey in our preliminary experiments. Note that although the three prey types differ in size and therefore subtend different viewing angles at the mantis eye, all are well above the size threshold for eliciting the maximum tracking and striking behavior (Prete et al. 2011) (e.g. the bee and mealworm subtends approximately 30 and 90 degrees of visual angle in length and 10 and 5 degrees in width, respectively, when located at 20 mm from the centre of the mantis’s view). The vertical position of the prey was adjusted to the centre of the mantis’s head. The mantises were surrounded with white walls in order to prevent any visual distraction, and their behaviour was monitored using a video camera (HDR-XR520V, SONY) placed above the apparatus.

### Protocol

**Fig. 3:**
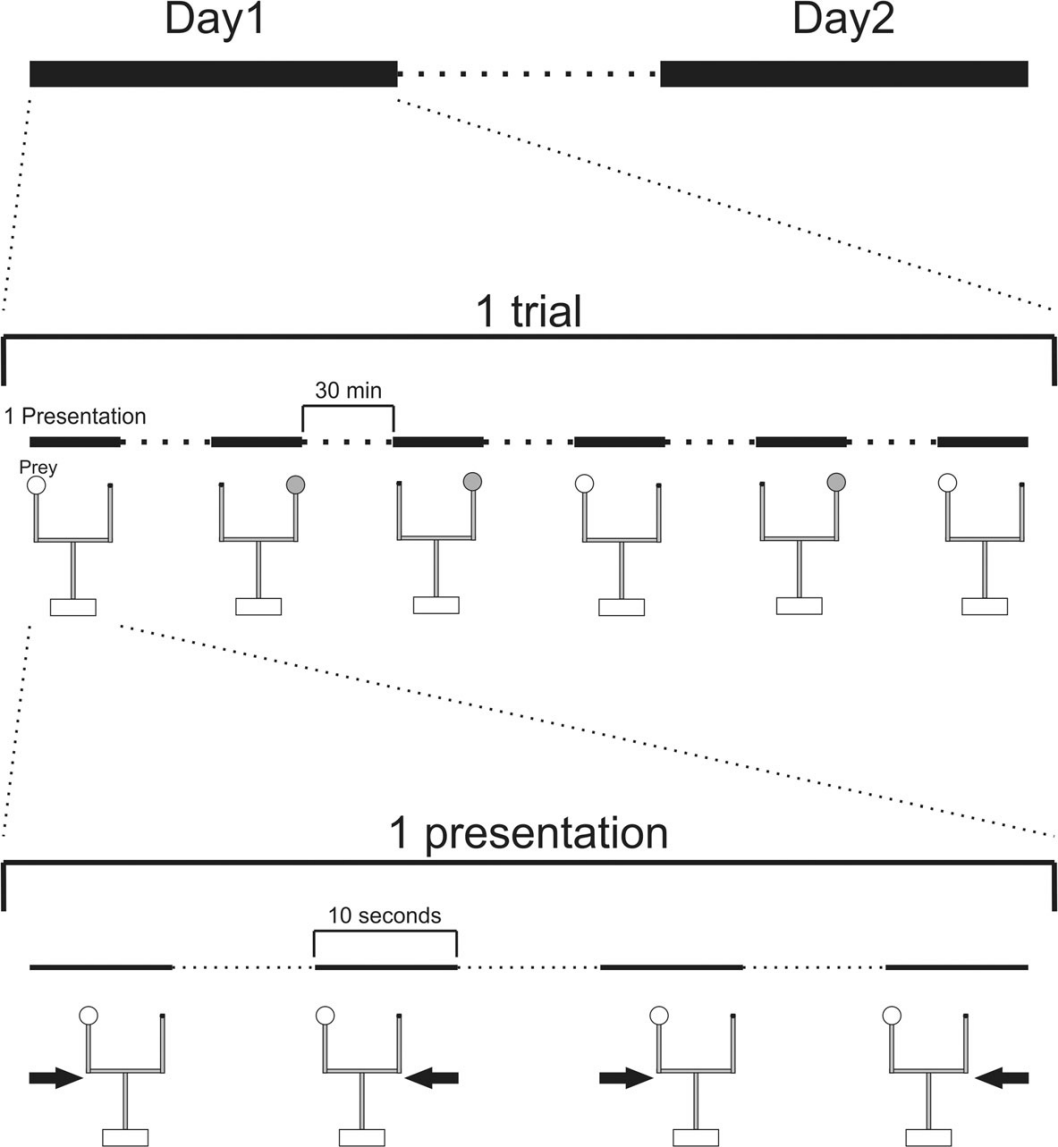
**Protocol of presentation.** During a session, the mantises received a trial every day. A trial consisted of six presentations: three for each of two prey species with a random order and an interval of 30 min between each prey. A presentation consisted of mechanically moving the prey in front of a mantis and wait for 10 s for an attack before moving the prey away. This sequence of prey movements was repeated four times or stopped after an attack

The protocol consisted of an acclimation session (during 3 days) followed by one day off and a learning session (during 6 days). In a session, the mantises received a trial per day that consisted of three presentations for each of two prey species (six presentations in total) with an interval of 30 min between each prey (see Fig. 3), as the female mantises showed that they are able to attack and eat about 8 mealworms per day (Carle et al. 2015). The presentation order was randomly determined for each individual. A presentation consisted of mechanically moving the prey in front of a mantis, waiting 10 s for an attack, and then moving the prey away. This sequence of prey movements was repeated four times during each presentation, until the mantis attacked the prey. For each trial, we measured the numbers of prey attacked and eaten.

During the acclimation session, both of the two prey species were injected in their abdomen and thorax with and coated by 100 μl of distilled water to ensure that the prey have the same shape after injection and no movements (due to our method of injection) as during the learning phase. The purpose of this session was to ensure that the mantises acclimated to the apparatus, and to investigate the preference of mantises for prey species. During the learning session, the prey were injected with a 100 μl solution of either 500 mM denatonium benzoate (Tokyo Chemical Industry, Japan) or distilled water. The concentration of denatonium benzoate (DB) was chosen based on our previous work showing that female mantises show aversive behaviour at this concentration (Carle et al. 2015). To examine the effects of prey type on avoidance learning, two different treatments (combinations of prey species and solutions) were used. For example, in the cricket/worm condition, half of mantises received crickets injected with water and mealworms injected with DB, and the other half of mantises received the reverse treatment (DB-injected crickets and water-injected mealworms).

### Data Analysis and Statistics

The data were plotted in excel files (Microsoft office, Microsoft corporation) and the statistical analysis was carried out using SPSS version 22 (IBM Corporation, www.ibm.com/software/analytics/spss). Because the data were not normally distributed (Shapiro-Wilk tests: *P* < 0.05), we employed generalized estimating equations (GEEs) that allowed us to use a more appropriate distribution to analyse these data (e.g. Poisson distribution with identity link function). We also reduced the risk of familywise errors by applying Holm-Bonferroni corrections.

First, we checked the response to the type of prey depending on the other prey presented in a pair (for example, we checked if there were significant differences in responses to crickets between the cricket/worm and cricket/bee conditions). Because we did not find any effects of the other prey (GEEs; for all values, χ^2^_2_ < 1.89, *P* > 0.05) or any interaction with another factor (for all values, χ^2^_1–2_ < 4.88, P > 0.05), we pooled the data together for the main analyses and provide the detailed experiments as supplementary files. Therefore, for the acclimation session data, we employed GEEs with types of prey (crickets, bees or mealworms) as categorical factor and days as ordinal factor, and adjusted α depending on Holm-Bonferroni corrections. For those of the learning session, we used GEEs using prey, days and bitterness (DB or water) as factors, and adjusted α to 0.0166 for the factor inducing the most significant result, to 0.025 for the factor inducing the second most significant result, and to 0.05 for the last factor.

### Ethical Notes

Because *T. aridifolia* is neither an endangered nor a protected species in Japan, no specific permission was required for collecting the oothecae. Moreover, our experimental procedure did not involve any physical damage. Through all the experiments, in total 13.6% of mantises (*N* = 6) died. However, because the mantises were freely able to ingest or reject prey and that bitter prey were easily detectable because they were coated with bitter solutions, it is unlikely that these deaths were due to bitter compounds ingested. During the manipulations, we took care to gently handle the mantises when we moved them between their cage and the experimental set-up. Furthermore, we anesthetized the mantises with cold, before fixing a piece of pin header. After these experiments, the mantises were used for physiological studies.

## Results

### Acclimation

**Fig. 4:**
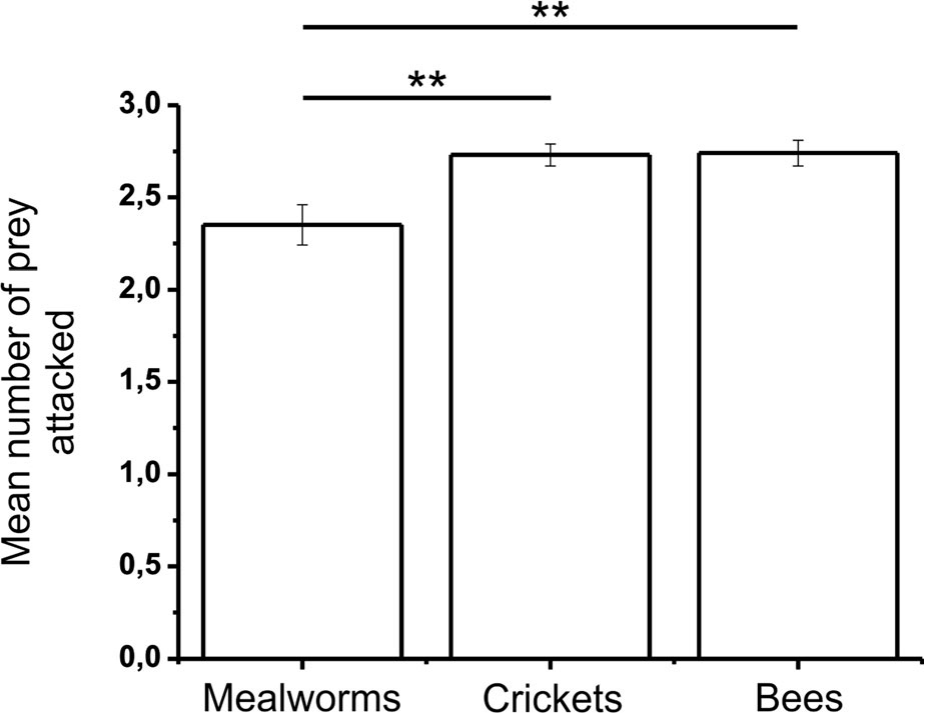
**Mean number per day of each type of prey attacked during the acclimation phase.** Statistics were done using Independent samples t-test and the results are represented on the graph (**: *p* < 0.01)

During the acclimation session, the mantises attacked and ate the prey from the first day, and seemed to show a preference for attacking bees and crickets rather than for mealworms (GEEs; χ^2^_2_ = 6.67, *P* = 0.036 > 0.025; Fig.4; Table 1). The mantises had a preference for attacking bees and crickets compared to mealworms (Independent samples t-test; t_104.13_ = −2.85, *P* < 0.01 and t_88.54_ = −2.96, P < 0.01 respectively). However, they attacked bees and crickets at the same levels (t_154_ = 0.11, *P* > 0.05). During the acclimation session, the amount of prey attacked did not vary over time: the number of mealworms, crickets and bees that were attacked stayed constant across the days, as well as the number of prey eaten (GEEs; for all values, χ^2^_2_ < 2.82, P > 0.05). In addition, the preference for attacking and eating a certain type of prey did not change over days (for both values, χ^2^_4_ < 4.70, *P* > 0.05).

**Table 1:** Number of prey attacked during the acclimation session

	Mealworms		Crickets		Bees		Mean	
	Mean	SEM	Mean	SEM	Mean	SEM	Mean	SEM
Day1	2.40	0.23	2.89	0.06	2.79	0.10	**2.72**	**0.08**
Day2	2.35	0.17	2.61	0.12	2.75	0.15	**2.58**	**0.08**
Day3	2.30	0.19	2.68	0.10	2.67	0.13	**2.57**	**0.08**
Mean	**2.35**	**0.11**	**2.73**	**0.06**	**2.74**	**0.07**		

The values in bold are represented as mean ± SEM

### Learning – Number of Prey Attacked

The treatment of the prey (water-injected vs. DB-injected) affected the number of attacks during the learning session. We found that the prey were less frequently attacked when injected with DB than water (GEEs; χ^2^_1_ = 33.25, *P* < 0.001). Therefore, we separated and analyzed the data depending on the prey treatment.

**Fig. 5:**
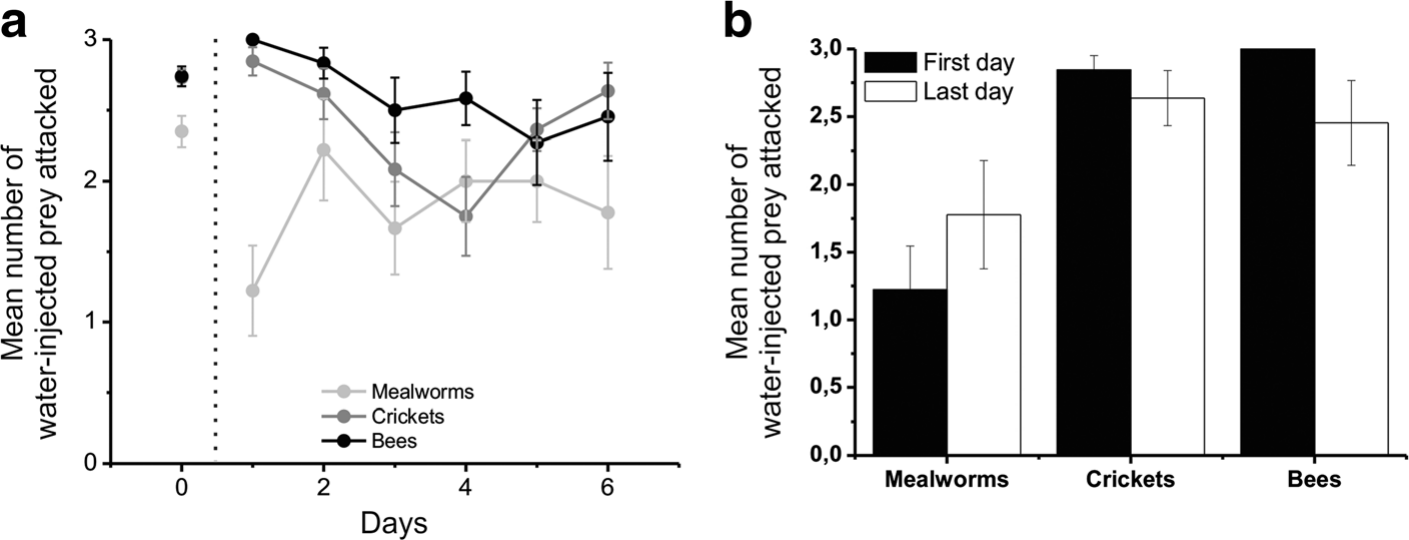
**Number of water-injected prey attacked during the learning phase. a:** Mean number ± SEM of prey attacked during the acclimation phase (points on the left) and within days during the learning phase that were injected with water. **b:** Mean number ± SEM of each type of prey injected with water that were attacked during the first (black columns) and last (white columns) days during the learning phase

In the case of water-injected prey (Fig. 5; Table 2), we found a similar pattern to that observed during the acclimation session. We found that the prey were not equally attacked (GEEs, χ^2^_2_ = 12.99, *P* = 0.002) and that this preference changed across days (χ^2^_10_ = 27.33, P = 0.002). However, we did not find any change in the overall rate of attacks across the days (χ^2^_5_ = 9.29, P > 0.05). We did not find any difference in the number of prey attacked between the first and the last day for the mealworms, crickets and bees (paired-sample t-tests; for all values, *t* < 1.74, *P* > 0.05; Fig. 4b). However, although the mantises kept constant their rate of attack on mealworms (GEEs, effects of days, χ^2^_5_ = 9.70, *P* > 0.05), we found that this rate changed for bees (χ^2^_5_ = 13.82, *P* < 0.05) and crickets (χ^2^_5_ = 69.81, *P* < 0.001) across the days (Fig. 4a). The rate of attack observed decreased within days and reached a peak at day 5 and 4 respectively for bees and crickets. Looking at the averaged number of prey attacked per day, we observed that the mantises attacked fewer mealworms than crickets (Independent samples t-test; t_96.47_ = −3.47, *P* < 0.01) and bees (t_94.29_ = −4.87, *P* < 0.001), and the number of attacks on crickets and bees was similar (t_140_ = −1.76, P > 0.05).

**Table 2:** Number of water-injected prey attacked during the learning session

	Mealworms		Crickets		Bees		Mean	
	Mean	SEM	Mean	SEM	Mean	SEM	Mean	SEM
Day1	1.22	0.32	2.85	0.10	3.00	0.00	**2.47**	**0.16**
Day2	2.22	0.36	2.62	0.18	2.83	0.11	**2.59**	**0.13**
Day3	1.67	0.33	2.08	0.26	2.50	0.23	**2.12**	**0.16**
Day4	2.00	0.29	1.75	0.28	2.58	0.19	**2.12**	**0.16**
Day5	2.00	0.29	2.36	0.15	2.27	0.30	**2.23**	**0.14**
Day6	1.78	0.40	2.64	0.20	2.45	0.31	**2.32**	**0.18**
Mean	**1.81**	**0.14**	**2.39**	**0.09**	**2.61**	**0.09**		

The values in bold are represented as mean ± SEM

**Fig. 6:**
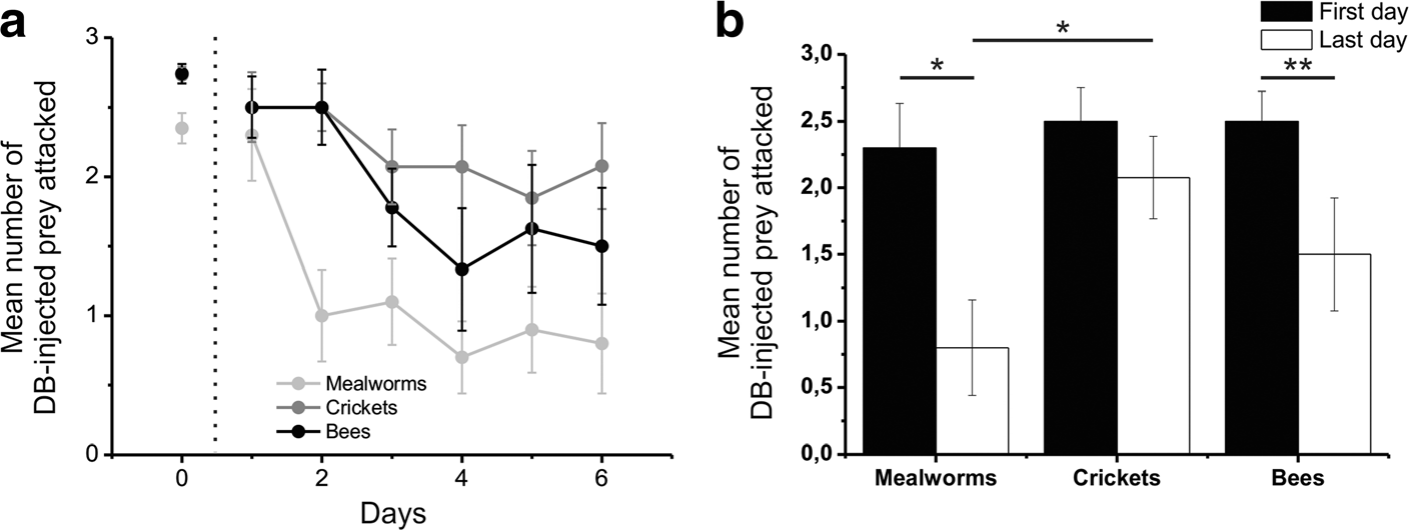
**Number of DB-injected prey attacked during the learning phase.** The legend is detailed in the Fig. 5. **b:** Statistics were done using Independent samples t-test and paired-sample t-tests. The results are represented on the graph (*: *p* < 0.05; **: p < 0.01)

In the case of DB-injected prey (Fig. 6; Table 3), the mantises reduced their attack on certain types of prey, but not on all (GEEs; χ^2^_2_ = 17.22, *P* < 0.001; Fig. 5a). Furthermore, the rate of attacks on DB-injected prey reduced across days (χ^2^_5_ = 30.06, P < 0.001), and this reduction varied depending on the prey (χ^2^_10_ = 30.72, P < 0.01). Basically, during the learning session, the mantises significantly reduced their attacks on bitter mealworms (GEEs; χ^2^_5_ = 32.47, P < 0.001), bitter bees (χ^2^_5_ = 19.23, P < 0.01) and bitter crickets (χ^2^_5_ = 11.55, *P* < 0.05) across the days. However, the reduction in attacks was greatest for bitter mealworms compared to the other prey: the mantises reduced their attacks by ~65% on bitter mealworms (t_9_ = 2.66, P < 0.05; Fig. 5b), by ~40% on bitter bees (t_7_ = 3.86, P < 0.01) and by ~17% on bitter crickets (t_12_ = 1.31, *P* > 0.05). During the last day, there were significantly fewer attacks on bitter mealworms than on bitter crickets (t_21_ = −2.70, P < 0.05), but no significant differences in the number of bitter bees attacked compared to bitter mealworms (t_16_ = −1.27, P > 0.05) or to bitter crickets (t_19_ = −1.21, P > 0.05).

**Table 3:** Number of DB-injected prey attacked during the learning session

	Mealworms		Crickets		Bees		Mean	
	Mean	SEM	Mean	SEM	Mean	SEM	Mean	SEM
Day1	2.30	0.33	2.50	0.25	2.50	0.22	**2.44**	**0.15**
Day2	1.00	0.33	2.50	0.17	2.50	0.27	**2.06**	**0.18**
Day3	1.10	0.31	2.07	0.27	1.78	0.28	**1.70**	**0.18**
Day4	0.70	0.26	2.07	0.30	1.33	0.44	**1.45**	**0.21**
Day5	0.90	0.31	1.85	0.34	1.62	0.46	**1.48**	**0.22**
Day6	0.80	0.36	2.08	0.31	1.50	0.42	**1.52**	**0.22**
Mean	**1.13**	**0.14**	**2.18**	**0.11**	**1.91**	**0.15**		

The values in bold are represented as mean ± SEM

### Learning – Proportion of Bitter Prey Eaten

**Fig. 7:**
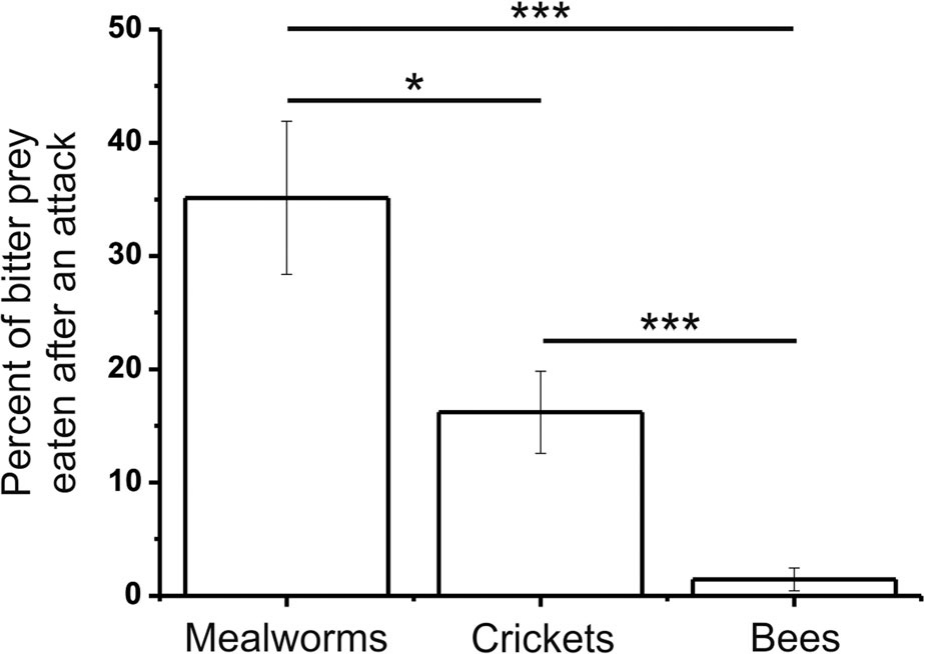
**Percent of bitter prey eaten after an attack for each type of prey during the learning phase.** Statistics were done using Independent samples t-test and paired-sample t-tests. The results are represented on the graph (*: p < 0.05; **: p < 0.01; ***: *p* < 0.001)

The mantises avoided ingesting bitter prey whatever the prey used (see supplementary files), but whether the bitter prey was ingested after an attack or not depended on the prey (Fig. 7). After a catch, the mantises ingested 35.1 ± 6.8% of bitter mealworms, 16.2 ± 3.6% of bitter crickets (Independent sample t-test; t_57.58_ = 2.43, P < 0.05), which was higher than 1.4 ± 1.0% of bitter bees (t_83.88_ = 3.96, P < 0.001). We noticed that the latter were almost totally rejected.

### Individuals’ Personality

**Fig. 8:**
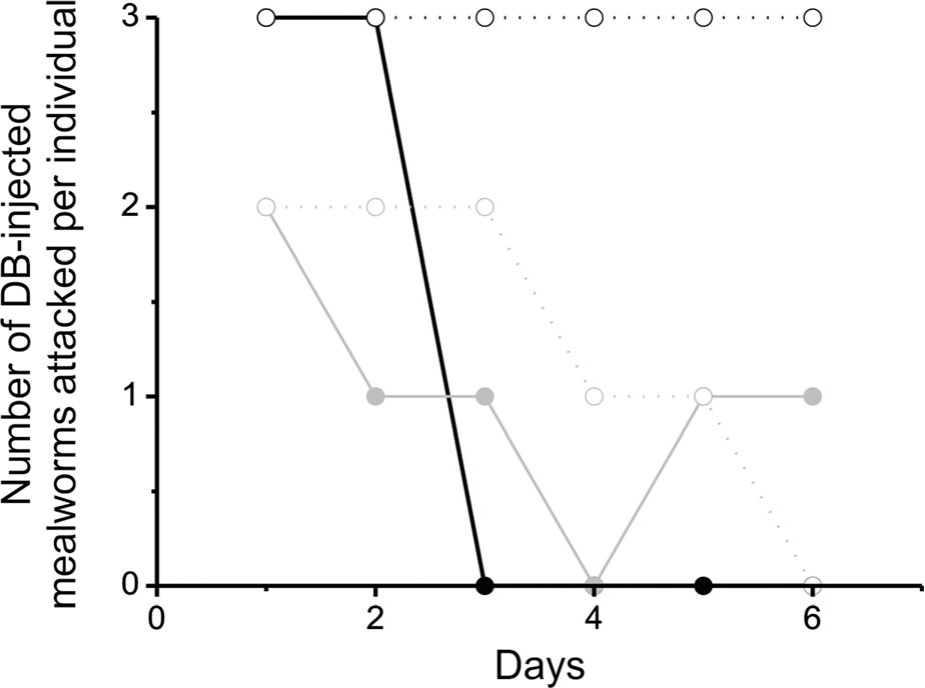
**Number of DB-injected bees that were attacked per day during the learning phase for 4 individuals.** Each symbol represents individual mantis

Although we did not detect any difference between the individuals during the learning session and their choice for prey (GLMs, individuals: χ^2^_36_ = 12.44, P > 0.05), the mantises did not show the same efficiency for learning (individuals: χ^2^_34_ = 73.89, P < 0.001). Looking at individuals’ rates of attack across the days, we noticed that some individuals continued to attack bitter prey whereas others learnt and reduced their attacks; Fig. 8 illustrates the range of responses with data from four intentionally selected individuals.

## Discussion

Using our protocol, the mantises learned to avoid novel bitter prey, and kept attacking familiar prey that were made bitter. However, they seemed to have specific constraints on avoidance learning compared to active foraging predators. While active foraging predators learn faster to avoid attacking bitter/toxic prey possessing conspicuous signals (Shettleworth 1972; Gittleman and Harvey 1980), we observed that using mealworms induced a stronger reduction of attacks in mantises than using honeybees. This rate of attack directly contrasts with their decision to ingest the prey: bitter mealworms were eaten more often, compared to bitter bees. Because the benefits from having warning coloration may differ depending on the aversive compound and on the predator, this finding might have a direct impact on mimic species and their evolution.

### Avoidance Learning in the Mantis

Avoidance learning for bitter food has been reported in many insects (e.g. in locusts: Bernays and Lee 1988) including mantises (Berenbaum and Miliczky 1984; Bowdish and Bultman 1993; Prudic et al. 2007). For example, Berenbaum and Miliczky (1984) have reported that mantises stop attacking milkweed bugs after several encounters. Furthermore, Prudic et al. (2007) tried to demonstrate that aversive learning is speeded and memory process lasts longer when conspicuous prey are used in mantises (*Tenodera aridifolia sinensis*) as observed in active foraging predators such as dragonflies (Kauppinen and Mappes 2003). However, in our study, conspicuous prey had different effects on avoidance learning in *Tenodera aridifolia*. Although mantises might not be able to detect color contrast (see Prudic et al. 2007; Fabricant and Herberstein 2014 for review), they are able to detect luminance contrast, and bees are perceived as more conspicuous than mealworms. Consequently, our results appeared inconsistent with those from these previous studies. Although we cannot exclude that this discrepancy might be due to differences in experimental design rather than biological reality, it may be explained by three possibilities: (1) visual preference for bees inhibited avoidance learning; (2) avoidance learning depended on post-digestive effects and compounds used as prey defense; (3) stronger association with bees compared to mealworms occurred during the acclimation session. Among these, our supplemental experiment refuted the last possibility. Indeed, bitter bees were still attacked even when the mantises did not have any experience with palatable bees during the acclimation phase (Supplemental Fig. 4).

One possibility is that visual preference for prey affected avoidance learning in the mantis. Preferences in food exist in many animals from humans to insects (Dethier 1954; Blaney 1981; Calef 1981; Simpson et al. 1988; Drewnowski 1997); and effects of food preference on avoidance learning have already been reported in insects such as in grasshoppers (e.g. Bernays and Lee 1988; Lee and Bernays 1990). It has been reported that taste avoidance is acquired less easily to more preferred food (Etscorn 1973). With mantises, preference may be correlated with and depend on visual stimuli as it has been shown by their rate of attack. It has been previously shown that the rate of attack in mantis depends on the speed, contrast, shape and size of visual cues that are used (Iwasaki 1990; Prete et al. 2002; Prete et al. 2011; Prete et al. 2013): for example, higher contrast visual cues induce higher rates of attack. The results of the acclimation session in our study suggest that food preference in our mantises seems to lean toward bees rather than mealworms. In other words, bees seem to have more effective luminance contrast and shape compared to mealworms for eliciting strikes in mantises; and such efficiency could result in a stronger inhibition of avoidance learning toward bees as found in the present study.

Another possibility would be that the avoidance learning depended on the compound used as a defense and/or its post-digestive effects. In the previous experiments with mantises, the authors used harmful compounds such as cardiac glycosides (Bowdish and Bultman 1993; Prudic et al. 2007) that are known to be bitter and toxic, or alkanes (Fabricant and Smith 2014) that are caustic but not bitter. In our study, the fact that we used DB known for being bitter but not toxic might be at the origin of such difference. However, ignoring the crickets because of their familiarity in our experimental conditions, we observed that the bitter bees were almost totally rejected after being attacked whereas the bitter mealworms were partially eaten. The fact that bitter mealworms were partially eaten seems correlated with the reduction of the rate of attack as days passed. In other words, the mantises might have developed avoidance for bitter mealworms because they ingested some. In this case, although it needs to be confirmed, it might be possible that DB would have post-digestive effects and that an effective learning depends on these post-digestive effects.

However, some mantises reduced their attack on bitter bees even if they did not ingest them as shown in Fig. 7, raising questions about the personality of individuals and their impact on evolution of prey’s defences (Royauté and Pruitt 2015). Furthermore, even if avoidance learning was reduced because of a lack of post-digestive effects, the mantises did not eat the bitter bees (~0%) after attacking them. From this observation, important questions remain concerning: 1) why bees are highly attacked and not eaten even though they were injected and coated with the same bitter solution as mealworms; 2) the cognitive capabilities in sit-and-wait predators facing different types of prey; and 3) the consequences of their cognitive capabilities on the selection pressure exerted by sit-and-wait predators on aposematic and mimic species.

Bees might be a more natural prey than mealworms for mantises. It might be interesting to determine how shape or luminance contrast affects the rate of attack in our experiment. A simple way would be to paint mealworms or bees in different colors. In addition, other experiments using different natural prey might help to elucidate food preference in mantises.

### Low Learning Efficiency in Sit-and-Wait Predators

Sit-and-wait predators might possess “limited” learning capabilities compared to active foraging predators. Low efficiency in discrimination learning has been already reported in several sit-and-wait spiders (e.g. Malcolm 1986; Toft and Wise 1999). Although hunting spiders shows avoidance learning capabilities, Jakob and Long (2016) pointed out that showing learning processes in spiders in laboratory conditions is not so easy and requires specific conditions (Jakob and Long 2016); for example a simple change of environment may abolish what they have learnt about the palatability of prey (Skow and Jakob 2006). In mantises, many studies on visual recognition of prey in mantis may suggest low learning efficiency, although learning processes have been reported (see Maldonado 1972). For example, when mantises are presented with prey-like stimuli on a computer screen without rewarding (for examples Yamawaki 2000; Prete et al. 2002; Yamawaki 2003; Prete et al. 2011), the mantises continue to attack even over a period of several days (e.g. Baum et al. 2014). Such low learning efficiency might present advantages for sit-and-wait predators, especially, the mantis. Indeed, catching every prey would be a beneficial strategy because of uncertainty in encountering another prey due to a sit-and-wait strategy. By holding prey with its forelegs and gradually eating the prey by chipping away at it, the mantis has enough time to find edible parts of prey and reject unpalatable parts/prey (Reitze and Nentwig 1991). Furthermore, by catching a prey, the mantises still do not lose any opportunity to find another prey because they can still detect and catch prey during eating (personal observation).

### Consequences on the Evolution of Warning Colorations

Our results might provide new insights on the factors affecting evolution of warning colorations (Bates 1861; Poulton 1890). In the wild, aposematic species differ in their defensive strategies (e.g. alkanes, cardiac glycosides, pyrazines) and their conspicuousness (Endler and Mappes 2004). As Endler and Mappes (2004) argue, certain species are ‘well-defended’ and ‘weakly conspicuous’. Our results on praying mantises are in accordance with the evolution of different forms of aposematism: the compound and/or its ingestion seems the main factor driving the learning processes. Therefore, although conspicuousness favours learning and memory processes, it may present a disadvantage in the case of sit-and-wait predators if these ones are not harmed by the compound or if they show low-efficiency learning for conspicuous stimuli, as our findings for the bees suggest. As a consequence, Batesian mimics, which are conspicuous without having harmful defences, might be under this additional pressure: they do not benefit from “taste-reject behavior” (Berenbaum and Miliczky 1984), and from learning processes in mantises and maybe in sit-and-wait predators in general.

## Electronic supplementary material

ESM 1 (DOCX 101 kb)

ESM 2 (JPEG 1998 kb)

High resolution image (TIFF 17746 kb)

ESM 3 (JPEG 2099 kb)

High resolution image (TIFF 26563 kb)

ESM 4 (JPEG 1995 kb)

High resolution image (TIFF 15859 kb)

ESM 5 (JPEG 2021 kb)

High resolution image (TIFF 19965 kb)
