# Supplementary material for: Aversive Learning in the Praying Mantis (Tenodera aridifolia), a Sit and Wait Predator
Source: J Insect Behav. 2018 Feb 22;31(2):158–75. doi: 10.1007/s10905-018-9665-1 (PMC5882761; doi:10.1007/s10905-018-9665-1)
Supplement: Supplementary file 1 — (DOCX 101 kb) [file 10905_2018_9665_MOESM1_ESM.docx]

**Methods**

*Effect of experience*

In addition, in order to prevent any effect of experience that could have occurred during the training session and induced biases in our study, we conducted another experiment (N=8) presenting only crickets during the training session, and bees/mealworms during the learning session. In other words, we did not present the same prey type during both training and learning sessions.

**Results**

Denatonium benzoate-injected prey

*Acclimation*

Looking at the individual experiments: we also did not observe any main effect of days (days: for all values, χ^2^_2_<4.62, P>0.05) or any interaction between days and type of prey (for all values, χ^2^_2_<5.73, P>0.05) for the numbers of prey attacked and eaten. Here again, the mantises showed a preference for bees and crickets rather than mealworms: there was a significant effect of the type of prey when mealworms were used (for all values, χ^2^_1_>7.32, P<0.01). The mantises attacked (Wilcoxon tests; for both values: z<-1.90, p<0.057) and ate (Wilcoxon tests; for both values: z<-1.97, p<0.049) crickets and bees more frequently than mealworms (Fig. 2). In addition, when bees and crickets were simultaneously presented, we did not observe any preference between these species in term of attack (z=-0.97, p=0.319) nor ingestion (z=-0.91, p=0.362).

*Learning – number of prey attacked*

During the learning sessions, the mantises that received bitter mealworms reduced their attack on mealworms whereas those receiving bitter crickets or bees still continued to attack these prey at a high rate (Fig. 3). Although there was no main effect of treatments (for all values: χ^2^_1_<0.19, P>0.05) for each condition, we found a significant interaction between treatments and days for the cricket/worm and bee/worm conditions (for all values: χ^2^_5_>18.13, P<0.01), but not for the cricket/bee condition (χ^2^_5_=3.13, P=0.679). In addition, we found a significant interaction between treatments and bitterness in the bee/worm condition (χ^2^_1_=6.66, P<0.01), and a tendency in the cricket/worm condition (χ^2^_1_=5.57, P=0.018) but not for the cricket/bee condition (χ^2^_1_=0.75, P=0.386).

Consequently, we separated and analyzed the data for each treatment in the cricket/worm and bee/worm conditions, and we found differences between the treatments. We found a main effect of bitterness (for all values: χ^2^_1_=5.42, P<0.025) and an interaction between days and bitterness (for all values: χ^2^_3_>10.35, P<0.025) when the mantises received bitter mealworms. In this case, the mantises attacked bitter mealworms less frequently than water-injected prey and reduced their attack on bitter mealworms during the session. In contrast, the mantises that received bitter bees or bitter crickets attacked bitter prey as much as water-injected prey (bitterness: χ^2^_1_<0.44, P>0.05). However, we have to report that we noticed a significant interaction between days and bitterness for the mantises that received bitter bees (χ^2^_3_=51.40, P<0.001). In this case, the mantises slightly reduced their attack on bitter bees and increased their attack on water-injected mealworms during the session.

*Learning – number of prey eaten*

The mantises avoided the ingestion of bitter prey in every condition (effect of bitterness: for all values, χ^2^_1_>15.10, P<0.001; Fig 4a). In addition, we found an almost significant effect of the treatments (for all values: χ^2^_1_>4.09, 0.014<P<0.05), and we decided to compare the results obtained for each treatment. In this condition, we observed that the mantises ate bitter bees less frequently than bitter mealworms and crickets (Mann-Whitney tests: bee/worm, U=126, z=-3.07, p=0.002; cricket/bee, U=635, z=-2.68, p=0.007), and bitter mealworms were eaten more frequently than bitter crickets (cricket/worm, U=211.5, z=-1.98, p=0.048; Fig. 4b). This tendency of reluctance to consume bitter bees was observed from the first day although it was not systematically significant (bee/worm, U=1.5, z=-1.76, p=0.08; cricket/bee, U=8.5, z=-2.41, p=0.021).

*Effect of experience*

Receiving only crickets during the training phase, and then bees and mealworms during the learning phase did not affect the mantises' avoidance for attacking bitter bees. Although there was no main effect of treatment (χ^2^_1_=1.41, P=0.235), we found a significant interaction between treatments and bitterness (χ^2^_1_=17.61, P<0.001), but not between treatments and days (χ^2^_5_=8.71, P=0.121). Once more, we separated the data depending on the treatment, and we found that the individuals that received bitter mealworms reduced their attacks on bitter mealworms (bitterness: χ^2^_1_=12.81, P<0.001; days: χ^2^_5_=88.86, P<0.001; interaction between days and bitterness: χ^2^_5_=380.51, P<0.001). In this case, we also found main effects of bitterness (χ^2^_1_=5.06, P=0.024) and days (χ^2^_5_=9.51, P<0.023) for the individuals receiving the reversed treatment. However, the group that received bitter bees still attacked more bees (day #6: 2.5 ± 0.29) than water-injected mealworms (day #6: 2 ± 0.58) at the end of learning, although they could not eat and associate potential nutritive values from bees as they were rejected from the first day (see below).

Here also, the mantises avoided ingesting bitter prey (χ^2^_1_=195.85, P<0.001; Fig. 6c), and we also observed a total reluctance towards eating bitter bees compared to eating bitter mealworms (U=132, z=-4.11, p<0.001; Fig. 6d).

Supplemental figure legends

Supplementary Figure 1:

**Number of each type of prey attacked (a) and eaten (b) during the acclimation phase for the different conditions:** cricket/worm (left), bee/worm (middle), cricket/bee (right). Boxes show the 25th and 75th percentiles and bisecting lines show the median value. Whiskers indicate the 5th and 95th percentiles. The mean value is also indicated (open circles). Statistics were done using Wilcoxon tests and the results are represented on each graph: p < 0.05; **: p < 0.01; ***: p < 0.001; as well as the number of individuals used.

Supplementary Figure 2:

**Number of prey attacked during the learning phase. a:** Mean number ± SEM of prey attacked within days that were injected with either water or DB for the different conditions: cricket/worm (left), bee/worm (middle), cricket/bee (right). **b:** Mean number ± SEM of each type of prey injected with either water or DB that were attacked within days for each treatment of mantises: treatment 1 (top) and treatment 2 (bottom).

Supplementary Figure 3:

**Number of prey eaten during the learning phase. a:** Mean number ± SEM of prey eaten within days that were injected with either water or DB for the different conditions (see figure 2 for details). **b:** Mean and median numbers of bitter prey eaten for each treatment of mantises during the entire learning session. Statistics were done using Mann-Whitney tests and the results are represented on each graph as well as the number of individuals used.

Supplementary Figure 4:

**Number of prey attacked and eaten during the learning phase when the mantises received only crickets during the acclimation phase. a-b:** See figure 3 for details. **c-d:** See figure 4 for details.
