# Supplementary figures and images for: Aversive Learning in the Praying Mantis (Tenodera aridifolia), a Sit and Wait Predator
Source: J Insect Behav. 2018 Feb 22;31(2):158–75. doi: 10.1007/s10905-018-9665-1 (PMC5882761; doi:10.1007/s10905-018-9665-1)

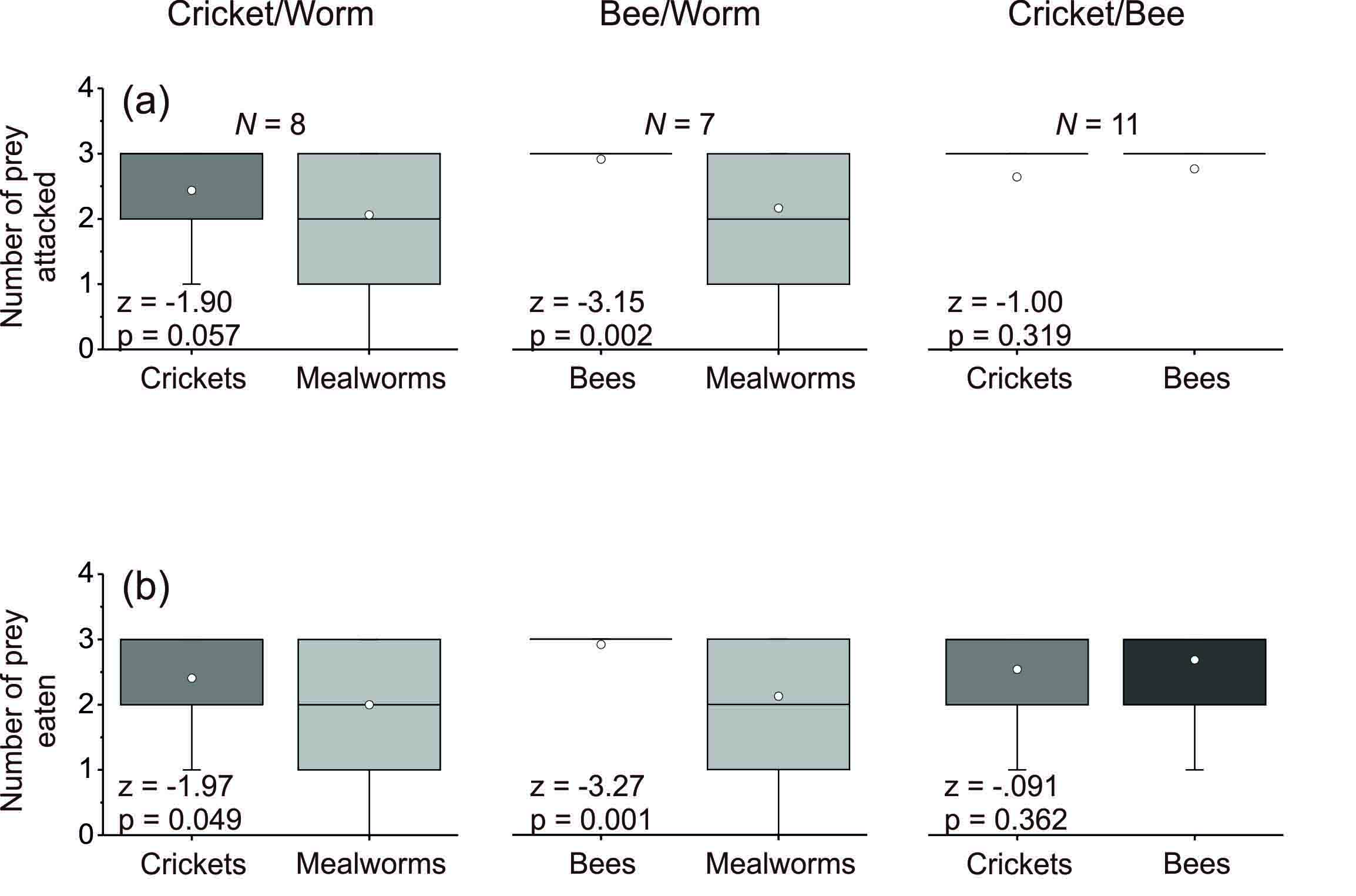

Supplement: Supplementary file 2 — (JPEG 1998 kb) [file 10905_2018_9665_Fig9_ESM.jpg]

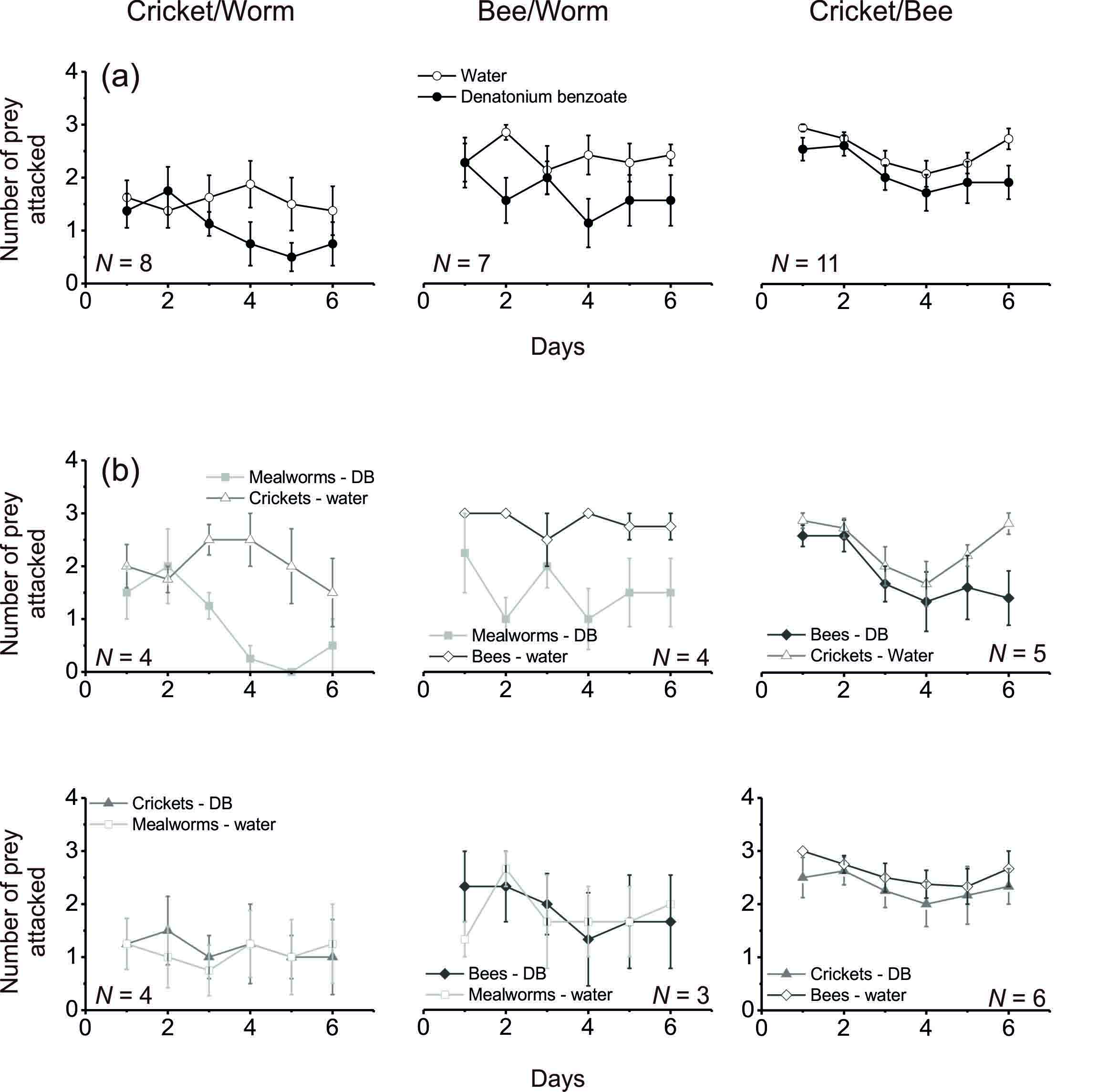

Supplement: Supplementary file 4 — (JPEG 2099 kb) [file 10905_2018_9665_Fig10_ESM.jpg]

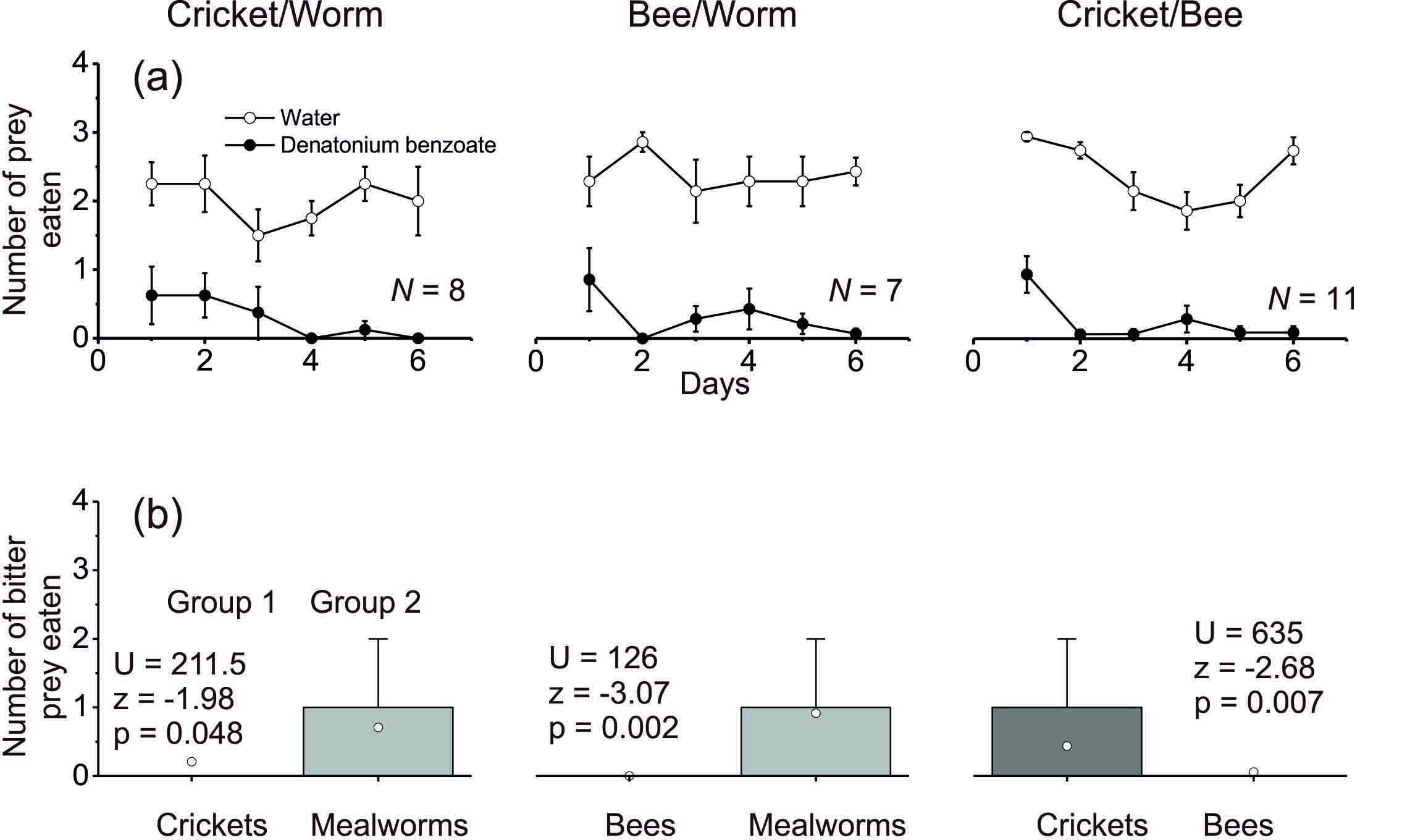

Supplement: Supplementary file 6 — (JPEG 1995 kb) [file 10905_2018_9665_Fig11_ESM.jpg]

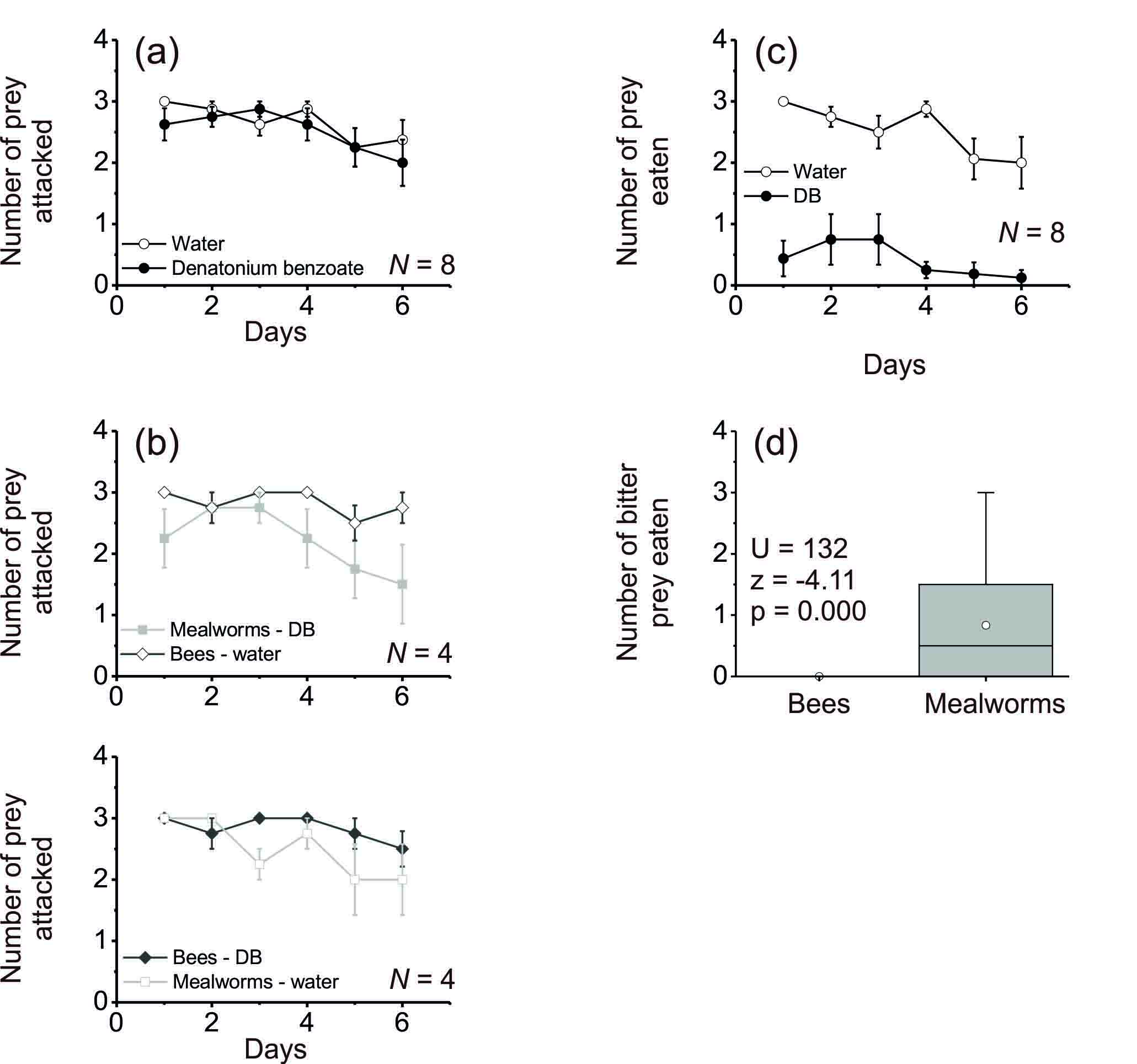

Supplement: Supplementary file 8 — (JPEG 2021 kb) [file 10905_2018_9665_Fig12_ESM.jpg]
